# Racial disparities in HIV incidence and PrEP non-adherence among gay, bisexual and other Men who have Sex with Men (MSM) and transgender women using oral PrEP in Brazil: Results from the ImPrEP study

**DOI:** 10.1016/j.bjid.2026.104614

**Published:** 2026-01-29

**Authors:** Lucilene Araujo Freitas, Carolina Coutinho, Debora Castanheira, Ronaldo Ismerio, Pedro Leite, Iuri C. Leite, Marcelo Cunha, Josias Freitas, Toni Araujo, Laylla Monteiro, Brenda Hoagland, Mayara S.T. Silva, Marcos Benedetti, Cristina Pimenta, Beatriz Grinsztejn, Valdilea G. Veloso, Thiago S. Torres

**Affiliations:** a Instituto Nacional de Infectologia Evandro Chagas, Fundação Oswaldo Cruz (INI-Fiocruz), Rio de Janeiro, RJ, Brazil; b Escola Nacional de Saúde Pública Sérgio Arouca, Fundação Oswaldo Cruz (ENSP-Fiocruz), Rio de Janeiro, RJ, Brazil

**Keywords:** Race, Racism, Discrimination, Homophobia, Sexual and gender minorities, LBTQIAPN+, Latin America

## Abstract

**Introduction:**

Access to health services may affect PrEP uptake, adherence, and persistance, especially among persons affected by structural inequities. We assessed the HIV incidence and factors associated with PrEP non-adherence among men who have sex with men (MSM) and transgender women in Brazil, focusing on potential racial disparities.

**Material and methods:**

ImPrEP was a prospective, single-arm, open-label, PrEP implementation study that enrolled 9509 MSM and transgender women in Brazil, Mexico and Peru (February 2018 ‒ June 2021). Participants received oral PrEP with tenofovir disoproxil fumarate and emtricitabine at the enrollment visit and quarterly thereafter. This analysis was restricted to data from 14 HIV/STI clinics in 11 cities from Brazil. We calculated the HIV incidence per 100 person-years using the Poisson model according to race. We used adjusted logistic regression models to identify factors associated with PrEP non-adherence (medication possession rate < 0.6) for White and Black/Pardo participants separately. Trial registration: UTN U1111-1217-6021

**Results:**

Of 3928 participants, 1868 (47.6%) self-identified as White, 1410 (35.9%) *Pardo*, 595 (15.1%) Black, 42 (1.1%) Asian, and 12 (0.3%) Indigenous. PrEP non-adherence was higher among Black (26.2%) and *Pardo* (24.2%) compared to White (18.7%) participants (*p* < 0.0001). Among participants with PrEP non-adherence, HIV incidence was higher among Black (2.16 [95% CI: 0.54‒8.63]) than *Pardo* (1.49 [95% CI: 0.48‒4.62]) and White (1.00 [95% CI 0.25‒4.01]). In multivariable models, participants aged 18‒30 years, self-identifying as transgender women, and reporting lower number of sex partners had increased odds of PrEP non-adherence among White and Black/*Pardo* participants. Lower education and transactional sex were associated with increased odds of PrEP non-adherence among Black/*Pardo*, but not among White participants.

**Conclusions:**

Higher HIV incidence and PrEP non-adherence among racially and ethnically marginalized groups, such as Black and *Pardo* Brazilians, highlights the impact of structural racism on health outcomes. The implementation of public policies to reduce racial and social inequities in HIV prevention must be prioritized in Brazil.

## Introduction

Despite major advances in prevention and treatment strategies in recent decades, HIV remains a major global public health problem. In 2024, there were 1.3 million new HIV acquisitions worldwide.^1^ In contrast with other regions, the HIV incidence continues to increase in Latin America since 2010.^2^ The HIV epidemic in the region is concentrated in certain populations, such as gay, bisexual and men who have sex with men (MSM) and transgender women.^2^, ^3^, ^4^ In Brazil, 39,216 new HIV acquisitions were detected in 2024, with a majority among Black and *Pardo* (Brazil’s official term for admixed populations) Brazilians.^5^

Pre-Exposure Prophylaxis (PrEP) has emerged as a critical tool in the efforts to prevent HIV. PrEP implementation has led to significant population-level reductions in HIV incidence highlighting the importance of this strategy in a global context, particularly among minority populations.^6^ Gender, sexual and racial minorities frequently experience increased vulnerability to HIV due to insufficient access to appropriate health services.^7^ The interplay of gender, sexual orientation, race, socioeconomic inequalities, and HIV incidence is complex and multifaceted, reflecting a system of inequality rooted in broader social structures.^7^

In Brazil, racial disparities in health have historical roots. As a result, racially and ethnically marginalized groups, such as Black, *Pardo* and Indigenous Brazilians, experience difficulties in accessing health care services and poorer health outcomes.^8^ Since the 1990s, race has been included in official data collection in Brazil. In 2005, the Brazilian Ministry of Health’s National HIV Program systems began to incorporate race information into their records. Nonetheless, it was not until 2017 that the collection of race data became a mandatory requirement within the information systems of the Brazilian Unified Health System (*Sistema Único de Saúde* [SUS]).^9^ Consequently, prior to this key milestone, it was more challenging to present and analyze health data in Brazil through a racial perspective.

In 2024, Black or *Pardo* persons accounted for 62% of new HIV cases in Brazil.^5^ Black and *Pardo* persons accounted for 71% of AIDS cases among children under 14 and 64% among those aged 15 to 29 years.^8^ In 2012, 51% of those who died from AIDS in Brazil were Black or *Pardo*, a proportion that increased to 62% in 2024.^5^ A recent cohort study that evaluated 28.3 million individuals for over a 9-year in Brazil found that self-identifying as Black was associated with a higher AIDS incidence (RR = 1.53; 95% CI: 1.45–1.61), mortality (RR = 1.69; 95% CI: 1.57–1.83) and case-fatality rates (RR = 1.16; 95% CI: 1.03–1.32), compared to White.^10^

Racial inequality, combined with the health disparities that affect LGBTQIAPN+ populations, exacerbates the vulnerability of these populations to several health outcomes, including HIV. In a recent study that included 8,463 Brazilian MSM, transgender and non-binary persons, Black participants reported experiencing higher levels of discrimination compared to their White counterparts.^11^ This finding highlights the greater burden of discrimination considering the intersection of gender, sexual orientation and race. Thus, understanding the role of racial disparities among LGBTQIAPN+ populations is essential for developing more effective and equitable health policies, as well as for designing prevention and treatment strategies that take these factors into account.

Although the state of the art on PrEP care continuum is increasing, significant gaps remain regarding the intersections of race and PrEP in low- and middle-income countries. In this context, this study aimed to evaluate the incidence of HIV and identify factors associated with PrEP non-adherence among MSM and transgender women attending PrEP services in Brazil, focusing on potential racial disparities.

## Material and methods

### Study design

ImPrEP was a prospective, single-arm, open-label, multicenter study of oral PrEP implementation conducted between February 2018 and June 2021 in Brazil, Peru, and Mexico. The main results of the study, as well as details of the study protocol, have been previously published.^12^, ^13^, ^14^ ImPrEP enrolled 9,509 cisgender MSM and transgender women aged 18-years or older who had tested HIV-negative and reported at least one PrEP eligibility criteria at the time of the study: condomless anal sex, anal sex with partner(s) living with HIV, rectal or urethral gonorrhea, chlamydia or syphilis and transactional sex (all related to 6-months prior to enrollment).

Data were collected on demographics, sexual behavior, substance use, and the main reason to attend the service. Follow-up visits were scheduled at week 4 and then quarterly until study termination in June 2021. At enrollment, participants received 30 tablets of tenofovir disoproxil fumarate (300 mg) co-formulated with emtricitabine (200 mg). At each subsequent visit, participants received medication refills according to the next scheduled visit interval. HIV testing was performed at all study visits using rapid tests according to the Brazilian algorithm.^15^

For this analysis, we only included participants from Brazil (*n* = 3928), as our aim was to investigate racial categories that are intrinsically connected to the country’s unique cultural context.^11^ Participants were recruited from 14 HIV/Sexually Transmitted Infections (STI) clinics in 11 municipalities in seven states: Brasília (Distrito Federal), Florianópolis (Santa Catarina), Manaus (Amazonas), Porto Alegre (Rio Grande do Sul), Recife (Pernambuco), Rio de Janeiro (*n* = 2; Rio de Janeiro), Niterói (Rio de Janeiro), Salvador (Bahia), Campinas (São Paulo), São Paulo (*n* = 3; São Paulo), and Santos (São Paulo).

### Socio-behavioral variables

Self-reported race was collected according to Brazilian census definition as Asian, Black, Indigenous, *Pardo* (mix-race), and White.^16^ Age was described as median and Interquartile Range (IQR) and in categorical intervals. Education level was categorized as primary (complete or incomplete), secondary (complete or incomplete) or more than secondary. The main reason for attending the service was stratified as seeking PrEP or other (seeking an HIV test, another health service or Post-Exposure Prophylaxis [PEP]). PEP use was assessed in relation to the previous 12-months (yes/no). We assessed the number of sex partners (median and IQR, categorized as < 5, 5‒10 or > 10), receptive condomless anal sex (yes/no), condomless anal sex with partner(s) living with HIV (yes/no/don't know) and transactional sex (yes/no). Binge drinking was defined as intake of five or more doses in a two-hour period (yes/no). Stimulant use was defined as the consumption of ecstasy, cocaine (powder, crack or paste), poppers or other inhalants. Questions on sexual behavior and substance use referred to the previous 6-months, except for number of sex partners (previous 3-months).

### Main outcomes

The HIV incidence was calculated by considering any HIV acquisition detected after enrollment, presented per 100 person-years, and stratified by race and PrEP non-adherence. PrEP non-adherence was assessed using the Medication Possession Rate (MPR), which was calculated by dividing the total number of tablets dispensed to the participant by the total number of days between enrollment (or previous visit) and the last visit. PrEP non-adherence was defined as MPR less than 0.6, following previous analyses.^12^^,^^13^ Participants with MPR of 0.6 or higher would possess enough PrEP pills to take at least four doses per week, which is considered sufficient for highly protective levels of tenofovir diphosphate.^17^

### Statistical analysis

We described the study population overall and according to the five categories of race (White, Pardo, Black, Indigenous and Asian). Associations were assessed using the chi-square test or Fisher’s exact test, as appropriate. Differences in medians were evaluated using the Kruskal-Wallis test. HIV incidence was estimated overall and according to PrEP non-adherence (yes/no) stratified by race. Incidence rates and their corresponding 95% Confidence Intervals were calculated using a Poisson regression model, with person-years of follow-up defined as the time from enrollment to the last study visit. Comparisons of population characteristics according to PrEP non-adherence were restricted to White and Black/*Pardo* participants, as the number of individuals who self-identified as Indigenous or Asian was too small for meaningful analysis. Baseline factors associated with PrEP non-adherence were subsequently identified using logistic regression models fitted separately for White and Black/*Pardo* participants. Variables with p-values < 0.25 in the unadjusted models were included in the multivariable analyses; only those remaining statistically significant at the 5% level were interpreted. All the analyses were conducted using SAS version 9.4.

## Results

Among 3,928 participants enrolled, 95.0% (*n* = 3,733) self-identified as cisgender man and 5.0% (*n* = 195) as transgender women (Table 1). The median age was 29-years (IQR: 24‒35), with the highest proportions in the > 30-years (1679; 42.7%) and 25‒30-years (1216; 31.0%) age groups. At the enrollment visit, most participants reported education higher than secondary (3101; 78.9%), attended the service seeking PrEP (3774; 96.1%), and reported no PEP use in the previous 12-months (2809; 71.5%). A total of 2115 (53.8%) participants reported having five or more sex partners, 2557 (65.1%) receptive condomless anal, and 397 (10.1%) engaged in transactional sex. Binge drinking was reported by 2566 (65.3%) participants, while 715 (18.2%) reported stimulant use.

**Table 1:** Characteristics of participants enrolled overall and according to race in the ImPrEP study, Brazil.

	Total	White	*Pardo*	Black	Asian	Indigenous	p-value
	*n* = 3928	*n* = 1868	*n* = 1410	*n* = 595	*n* = 42	*n* = 13	
	n (%)	n (%)	n (%)	n (%)	n (%)	n (%)	
**Gender**							0.0002^a^
Cisgender man	3733 (95.0)	1796 (96.1)	1312 (93.0)	574 (96.5)	40 (95.2)	11 (84.6)	
Transgender woman	195 (5.0)	72 (3.9)	98 (7.0)	21 (3.5)	2 (4.8)	2 (15.4)	
**Age (years)**							<0.0001^b^
Median (IQR)	29 (24‒35)	30 (25‒36)	28 (23‒34)	28 (24‒34)	27.5 (23‒35)	26 (23‒30)	<0.0001^c^
18‒19	122 (3.1)	36 (1.9)	65 (4.6)	19 (3.2)	2 (4.8)	0 (0.0)	
20‒21	269 (6.8)	94 (5.0)	118 (8.4)	51 (8.6)	3 (7.1)	3 (23.1)	
22‒24	642 (16.3)	259 (13.9)	260 (18.4)	112 (18.8)	9 (21.4)	2 (15.4)	
25‒30	1216 (31.0)	565 (30.2)	443 (31.4)	193 (32.4)	10 (23.8)	5 (38.5)	
> 30	1679 (42.7)	914 (48.9)	524 (37.2)	220 (37.0)	18 (42.9)	3 (23.1)	
**Education**							<0.0001^a^
Primary	52 (1.3)	18 (1.0)	21 (1.5)	12 (2.0)	0 (0.0)	1 (7.7)	
Secondary	775 (19.7)	274 (14.6)	344 (24.4)	144 (24.2)	10 (23.8)	3 (23.1)	
More than secondary	3101 (78.9)	1576 (84.4)	1045 (74.1)	439 (73.8)	32 (76.2)	9 (69.2)	
**Main reason to attend the service**							0.78^a^
Seeking PrEP	3774 (96.1)	1789 (95.8)	1360 (96.5)	572 (96.1)	41 (97.6)	12 (92.3)	
Other	154 (3.9)	79 (4.2)	50 (3.5)	23 (3.9)	1 (2.4)	1 (7.7)	
**PEP use**^1^							0.65^a^
Yes	1119 (28.5)	522 (27.9)	399 (28.3)	180 (30.3)	15 (35.7)	3 (23.1)	
No	2809 (71.5)	1346 (72.1)	1011 (71.7)	415 (69.7)	27 (64.3)	10 (76.9)	
**Number of sex partners**^2^							<0.0001^a^
Median (IQR)	5 (2‒15)	5 (2‒12)	5 (2‒20)	5 (2‒15)	4 (2‒15)	8 (4‒20)	0.32^§^
< 5	1813 (46.2)	842 (45.1)	650 (46.1)	293 (49.2)	24 (57.1)	4 (30.8)	
5‒10	968 (24.6)	529 (28.3)	295 (20.9)	135 (22.7)	5 (11.9)	4 (30.8)	
> 10	1147 (29.2)	497 (26.6)	465 (33.0)	167 (28.1)	13 (31.0)	5 (38.5)	
**Receptive condomless anal sex**^2^							0.0060^a^
Yes	2557 (65.1)	1205 (64.5)	962 (68.2)	357 (60.0)	24 (57.1)	9 (69.2)	
No	1371 (34.9)	663 (35.5)	448 (31.8)	238 (40.0)	18 (42.9)	4 (30.8)	
**Condomless anal sex with partner(s) living with HIV**^2^							<0.0001^a^
Yes	824 (21.0)	382 (20.4)	302 (21.4)	130 (21.8)	8 (19.0)	2 (15.4)	
No	1144 (29.1)	614 (32.9)	352 (25.0)	159 (26.7)	17 (40.5)	2 (15.4)	
I don’t know	1960 (49.9)	872 (46.7)	756 (53.6)	306 (51.4)	17 (40.5)	9 (69.2)	
							
**Transactional sex**^2^							<0.0001^a^
Yes	397 (10.1)	133 (7.1)	185 (13.1)	70 (11.8)	5 (11.9)	4 (30.8)	
No	3531 (89.9)	1735 (92.9)	1225 (86.9)	525 (88.2)	37 (88.1)	9 (69.2)	
**Binge drinking**^2^							0.0076^a^
Yes	2566 (65.3)	1174 (62.8)	959 (68.0)	390 (65.5)	32 (76.2)	11 (84.6)	
No	1362 (34.7)	694 (37.2)	451 (32.0)	205 (34.5)	10 (23.8)	2 (15.4)	
**Stimulant use**^2^^,^^3^							0.0030^a^
Yes	715 (18.2)	376 (20.1)	240 (17.0)	91 (15.3)	5 (11.9)	3 (23.1)	
No	3213 (81.8)	1492 (79.9)	1170 (83.0)	504 (84.7)	37 (88.1)	10 (76.9)	
**PrEP non-adherence**^4^							<0.0001^a^
No	3053 (77.7)	1518 (81.3)	451 (75.8)	1040 (73.8)	10 (76.9)	4 (31.0)	
Yes	875 (22.3)	350 (18.7)	144 (24.2)	370 (26.2)	3 (23.1)	8 (19.0)	

1 Last 12-months.

2 Last 6-months.

3 Stimulant use was defined as use of any club drugs (e.g., ecstasy, LSD and GHB), cocaine (powder, crack, or paste), poppers or other inhalants.

4 PrEP non-adherence defined as MPR < 0·6, which is equivalent to less than four PrEP pills per week.

a Chi-Squared test.

b Fisher’s exact test with Monte Carlo approximation

c Kruskal-Wallis test for median.

Most participants self-identified as White (1868; 47.6%), followed by *Pardo* (1410; 35.9%), Black (595; 15.1%), Asian (42; 1.1%) and Indigenous (13; 0.3%). Compared to White, Black and *Pardo* participants were younger (aged 18‒30 years, White: 51.1%; *Pardo*: 62.8%; Black: 63.0%; *p* < 0.0001) and more reported secondary education or lower (White: 15.6%; *Pardo*: 25.9%; Black: 26.2%; *p* < 0.0001).

Overall, 875 (22.3%) participants exhibited an MPR less than 0.6 and were therefore considered with PrEP non-adherence. PrEP non-adherence was more frequent among Black (370; 26.2%) and *Pardo* (144; 24.2%) compared to White participants (350; 18.7%; *p* < 0.0001).

The HIV incidence was 0.36 (95% CI 0.24‒0.54) per 100 person-years, higher among those with PrEP non-adherence (1.40 [95% CI 0.67‒2.93] per 100 person-years) than PrEP adherence (0.28 [95% CI 0.17‒0.45] per 100 person-years) (Table 2). The HIV incidence was higher among Black and *Pardo* participants compared to White (White: 0.28 [95% CI 0.15‒0.55] per 100 person-years; *Pardo*: 0.43 [95% CI 0.23‒0.80] per 100 person-years; Black: 0.40 [95% CI 0.15‒0.16] per 100 person-years). Racial disparities on HIV incidence were more pronounced when considering only persons with PrEP non-adherence as follows: 1.00 [95% CI 0.25‒4.01] per 100 person-years among White participants; 1.49 [95% CI 0.48‒4.62] per 100 person-years among *Pard*o; and 2.16 [95% CI 0.54‒8.63] per 100 person-years among Black.

**Table 2:** HIV incidence overall and according to PrEP non-adherence stratified per race.

	Overall			PrEP non-adherence^a^					
				Yes MPR < 0.6			No MPR ≥ 0.6		
	HIV infection, N	Person-years of follow-up^b^	Incidence rate per 100 person-years (95% CI)	HIV infection, N	Person-years of follow-up^b^	Incidence rate per 100 person-years (95% CI)	HIV infection, N	Person-years of follow-up^b^	Incidence rate per 100 person-years (95% CI)
**Overall**	24	6577.81	0.36 (0.24‒0.54)	7	501.14	1.40 (0.67‒2.93)	17	6076.67	0.28 (0.17‒0.45)
**Race**									
White	9	3170.24	0.28 (0.15‒0.55)	2	199.47	1.00 (0.25‒4.01)	7	2970.78	0.24 (0.11‒0.49)
*Pardo*	10	2309.79	0.43 (0.23‒0.80)	3	201.13	1.49 (0.48‒4.62)	7	2108.67	0.33 (0.16‒0.70)
Black	4	1007.28	0.40 (0.15‒1.06)	2	92.70	2.16 (0.54‒8.63)	2	914.58	0.22 (0.05‒0.87)
Indigenous	0	20.30	0.00 (0.00‒14.76)	0	0.71	0.00 (0.00‒2676.06)	0	19.59	0.00 (0.00‒15.29)
Asian	1	70.20	1.42 (0.20‒10.11)	0	7.14	0.00 (0.00‒42.10)	1	63.06	1.59 (0.22‒11.26)

a PrEP non-adherence defined as MPR < 0·6, which is equivalent to less than four PrEP pills per week.

b Median length of follow-up (IQR): 1·99-years (IQR: 1·00‒2·49).

Characteristics of participants according to PrEP non-adherence (yes/no) stratified per race (White vs. Black/*Pardo*) are depicted on Table 3. Compared to White participants with PrEP non-adherence, Black/*Pardo* participants were frequently younger (18‒30 years: 73.6% vs. 68.0%; *p* = 0.054), had secondary education or lower (33.7% vs. 21.7%; *p* = 0.0003), reported condomless anal sex with partner(s) living with HIV (20.4% vs. 16.3; *p* = 0.024), transactional sex (17.5% vs. 11.1%; *p* = 0.0099) and binge drinking (69.1% vs. 60.0%; *p* = 0.0060). Compared to White participants with PrEP non-adherence, Black/*Pardo* participants were frequently younger (18‒30 years: 58.2% vs. 47.2%; *p* < 0.0001), had secondary education or lower (23.3% vs. 14.2%; *p* < 0.0001), reported more than 10 sex partners (32.1% vs. 26.1%; *p* < 0.0001), condomless anal sex with partner(s) with unknown HIV status (58.3% vs. 45.7%; *p* < 0.0001), transactional sex (11.1% vs. 6.2%; *p* < 0.0001) and stimulant use (16.1% vs. 19.6%; *p* = 0.013).

**Table 3:** Characteristics of participants according to PrEP non-adherence overall and stratified per race (White vs. Black/*Pardo*).

	PrEP non-adherence				PrEP adherence			
	MPR < 0.6				MPR ≥ 0.6			
	Overall	White	Black/*Pardo*	p-value	Overall	White	Black/*Pardo*	p-value
	864	350	514		3009	1518	1491	
**Gender**				0.47^a^				0.0093^a^
Cisgender man	785 (90.9)	321 (91.7)	464 (90.3)		2897 (96.3)	1475 (97.2)	1422 (95.4)	
Transgender woman	79 (9.1)	29 (8.3)	50 (9.7)		112 (3.7)	43 (2.8)	69 (4.6)	
**Age (years)**				0.054^a^				<0.0001^a^
18-24	321 (37.2)	114 (32.6)	207 (40.3)		693	275 (18.1)	418 (28.0)	
25-30	295 (34.1)	124 (35.4)	171(33.3)		906 (30.1)	441 (29.1)	465 (31.2)	
> 30	248 (28.7)	112 (32.0)	136 (26.4)		1410 (46.9)	802 (52.8)	608 (41.8)	
**Education**				0.0003^a^				<0.0001^a^
Primary	22 (2.5)	4 (1.1)	18 (3.5)		29 (1.0)	14 (0.9)	15 (1.0)	
Secondary	227 (26.3)	72 (20.6)	155 (30.2)		535 (17.8)	202 (13.3)	333 (22.3)	
More than secondary	615 (71.2)	274 (78.3)	341 (66.3)		2445 (81.3)	1302 (85.8)	1143 (76.7)	
**Main reason to attend the service**				0.64^a^				
Seeking PrEP	819 (94.8)	330 (94.3)	489 (95.1)		2902 (96.4)	1459 (96.1)	1443 (96.8)	0.32^a^
Other	45 (5.2)	20 (5.7)	25 (4.9)		107 (3.6)	59 (3.9)	48 (3.2)	
**Number of sex partners**^2^				0.40^a^				<0.0001^a^
< 5	432 (50.0)	169 (48.3)	263 (51.2)		1353 (45.0)	673 (44.3)	680 (45.6)	
5‒10	178 (20.6)	80 (22.9)	98 (19.1)		781 (26.0)	449 (29.6)	332 (22.3)	
> 10	254 (29.4)	101 (28.9)	153 (29.8)		875 (29.1)	396 (26.1)	479 (32.1)	
**Receptive condomless anal sex**^3^				0.77^a^				0.21^a^
Yes	543 (62.8)	222 (63.4)	321 (62.4)		1981 (65.8)	983 (64.8)	998 (66.9)	
No	321 (37.2)	128 (36.6)	193 (37.6)		1028 (34.2)	535 (35.2)	493 (33.1)	
**Condomless anal sex with partner(s) living with HIV**^3^				0.024^a^				<0.0001^a^
Yes	162 (18.8)	57 (16.3)	105 (20.4)		652 (21.7)	325 (21.4)	327 (21.9)	
No	242 (28.0)	115 (32.9)	127 (24.7)		883 (29.3)	499 (32.9)	384 (25.8)	
I don’t know	460 (53.2)	178 (50.9)	282 (54.9)		1474 (49.0)	694 (45.7)	780 (58.3)	
**Transactional sex**^3^				0.0099^a^				<0.0001^a^
Yes	129 (14.9)	39 (11.1)	90 (17.5)		259 (8.6)	94 (6.2)	165 (11.1)	
No	735 (85.1)	311 (88.9)	424 (82.5)		2750 (91.4)	1424 (93.8)	1326 (88.9)	
								
**Binge drinking**^3^								0.069^a^
Yes	565 (65.4)	210 (60.0)	355 (69.1)	0.0060^a^	1958 (65.1)	964 (63.5)	994 (66.7)	
No	299 (34.6)	140 (40.0)	159 (30.9)		1051 (34.9)	554 (36.5)	497 (33.3)	
**Stimulant use**^3^^,^^4^				0.077^a^				0.013^a^
Yes	170 (19.7)	79 (22.6)	91 (17.7)		537 (17.8)	297 (19.6)	240 (16.1)	
No	694 (80.3)	271 (77.4)	423 (82.3)		2472 (82.2)	1221 (80.4)	1251 (83.9)	

^1^ PrEP non-adherence defined as MPR < 0·6, which is equivalent to less than four PrEP pills per week.

2 Last 3-months.

3 Last 6-months.

4 Stimulant use was defined as use of any club drugs (e.g., ecstasy, LSD and GHB), cocaine (powder, crack, or paste), poppers or other inhalants.

a Chi-Squared test.

**Fig. 1:**
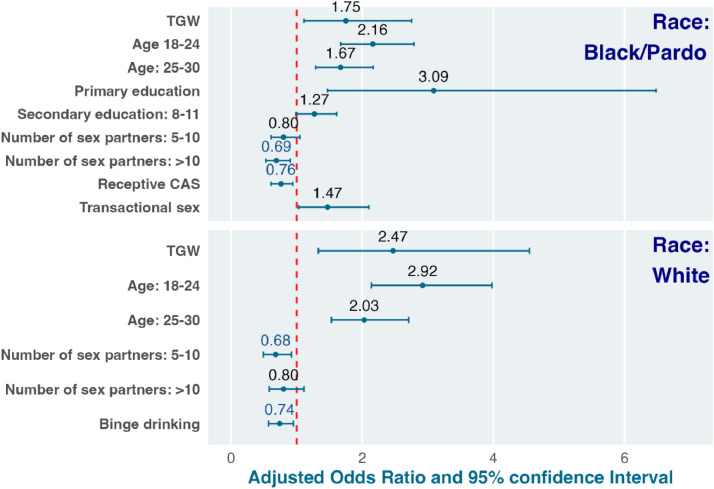
Factors associated with PrEP non-adherence among Black/*Pardo* and White participants. TGW: transgender women.

In multivariable analysis including only White participants, self-identifying as transgender woman (aOR = 2.47 [95% CI 1.33‒4.55]) compared to cisgender man and reporting younger age (18‒24 years: aOR = 2.92 [95% CI 2.14‒3.98]; 25‒30 years: aOR = 2.03 [95% CI 1.53‒2.71]) compared to > 30 years increased the odds of PrEP non-adherence (Table 4 and Fig. 1). Reporting 5‒10 sex partners (aOR = 0.68 [95% CI 0.49‒0.92]) compared to less than 5 and reporting binge drinking (aOR = 0.74 [95% CI 0.57‒0.95]) decreased the odds of PrEP non-adherence.

**Table 4:** Factors associated with PrEP non-adherence among participants self-identified as Black or *Pardo* and White.

	White				Black*/Pardo*			
	Univariable analyses		Multivariable analysis		Univariable analyses		Multivariable analysis	
	OR (95% CI)	p-value	aOR (95% CI)	p-value	OR (95% CI)	p-value	aOR (95% CI)	p-value
**Gender**								
Cisgender man	1.00				1.00		1.00	
Transgender woman	3.10 (1.91‒5.04)	<0.0001	2.47 (1.33‒4.55)	0.0038	2.22 (1.52‒3.24)	<0.0001	1.75 (1.11‒2.75)	0.015
**Age (years)**								
18‒24	2.97 (2.21‒3.98)	<0.0001	2.92 (2.14‒3.98)	<0.0001	2.21 (1.72‒2.84)	<0.0001	2.16 (1.67‒2.79)	<0.0001
25‒30	2.01 (1.52‒2.66)	<0.0001	2.03 (1.53‒2.71)	<0.0001	1.64 (1.27‒2.12)	0.0001	1.67 (1.29‒2.17)	0.0001
> 30	1.00		1.00		1.00		1.00	
**Education**								
Primary	1.36 (0.44‒4.16)	0.59	0.74 (0.22‒2.27)	0.62	4.02 (2.01‒8.06)	<0.0001	3.09 (1.47‒6.48)	0.0029
Secondary	1.69 (1.26‒2.28)	0.0005	1.18 (0.85‒1.63)	0.32	1.56 (1.24‒1.96)	0.0001	1.27 (0.99‒1.61)	0.056
More than secondary	1.00		1.00		1.00		1.00	
**Main reason to attend the service**								
Seeking PrEP	0.67 (0.40‒1.12)	0.13	0.74 (0.43‒1.27)	0.28	0.65 (0.40‒1.07)	0.088	0.70 (0.42‒1.18)	0.18
Other	1.00		1.00		1.00		1.00	
**Number of sex partners**^2^								
< 5	1.00		1.00		1.00		1.00	
5‒10	0.71 (0.53‒0.95)	0.021	0.68 (0.49‒0.92)	0.0014	0.76 (0.58‒1.00)	0.047	0.80 (0.61‒1.05)	0.12
> 10	1.02 (0.77‒1.34)	0.91	0.80 (0.58‒1.11)	0.18	0.82 (0.66‒1.04)	0.10	0.69 (0.53‒0.90)	0.0065
**Receptive condomless anal sex**^3^								
Yes	0.94 (0.74‒1.20)	0.64	NA	NA	0.82 (0.67‒1.01)	0.065	0.76 (0.61‒0.94)	0.0012
No	1.00		NA	NA	1.00		1.00	
**Condomless anal sex with partner(s) living with HIV**^3^								
Yes	0.76 (0.54‒1.08)	0.12	0.78 (0.54‒1.11)	0.17	0.97 (0.72‒1.31)	0.84	NA	NA
No	1.00		1.00		1.00		NA	NA
I don’t know	1.11 (0.86‒1.45)	0.42	1.22 (0.92‒1.61)	0.17	1.09 (0.86‒1.39)	0.47	NA	NA
**Transactional sex**^3^								
Yes	1.90 (1.28‒2.81)	0.0014	1.11 (0.66‒1.87)	0.69	1.71 (1.29‒2.25)	0.0002	1.47 (1.03‒2.10)	0.033
No	1.00		1.00		1.00		1.00	
**Binge drinking**^3^								
Yes	0.86 (0.68‒1.09)	0.22	0.74 (0.57‒0.95)	0.018	1.12 (0.90‒1.39)	0.32	NA	NA
No	1.00		1.00		1.00		NA	NA
**Stimulant use**^3^^,^^4^								
Yes	1.20 (0.90‒1.59)	0.21	1.29 (0.95‒1.74)	0.10	1.12 (0.86‒1.46)	0.40	NA	NA
No	1.00		1.00		1.00		NA	NA

OR, Odds Ratio; aOR, Adjusted OR; 95% CI, 95% Confidence Interval; NA, Not Applicable.

^1^ MPR ≥ 0·6 is equivalent to 4 PrEP pills per week.

2 Last 3-months.

3 Last 6-months.

4 Stimulant use was defined as use of any club drugs (e.g. ecstasy, LSD and GHB), cocaine (powder, crack, or paste), poppers or other inhalants.

In multivariable analysis including only Black/*Pardo* participants, self-identifying as transgender woman (aOR = 1.75 [95% CI 1.11‒2.75] compared to cisgender man, reporting younger age (18‒24 years: aOR = 2.16 [95% CI 1.67‒2.80]; 25‒30 years: aOR = 1.67 [95% CI 1.29‒2.17]) compared to > 30-years, having primary education (aOR = 3.09 [95% CI 1.47‒6.54]) compared to more than secondary education, and reporting transactional sex (aOR = 1.47 [95% CI 1.03‒2.09]) increased the odds of PrEP non-adherence. Reporting more than 10 sex partners (aOR = 0.69 [95% CI 0.53‒0.90]) compared to less than 5 and receptive condomless anal sex (aOR = 0.76 [95% CI 0.61‒0.94]) decreased the odds of PrEP non-adherence.

## Discussion

In the largest oral PrEP implementation study in Latin America, we found pronounced racial disparities in HIV incidence and PrEP non-adherence among MSM and transgender women attending PrEP services in Brazil. Among participants with PrEP non-adherence, HIV incidence was highest among Black participants (2.16 per 100 person-years; 95% CI: 0.54–8.63), followed by *Pardo* (1.49 per 100 person-years; 95% CI: 0.48–4.62) and White participants (1.00 per 100 person-years; 95% CI: 0.25–4.01). The proportion of participants with PrEP non-adherence was higher among Black (26.2%) and *Pardo* (24.2%) compared to White participants (18.7%). In multivariable analyses, transgender women, younger age, and behavior factors such as lower number of sex partners were identified as factors associated with PrEP non-adherence for White and Black/*Pardo* participants. Same associations were identified in previous analyses considering all participants from Brazil, Mexico and Peru included in the ImPrEP study.^12^^,^^14^ Among Black and *Pardo* participants, lower education and engagement in transactional sex were also associated with PrEP non-adherence. These findings underscore the need for targeted strategies to address both social and behavioral determinants that contribute to HIV vulnerability and PrEP non-adherence among marginalized groups.

Similar to our findings, emerging evidence highlights significant racial and ethnic disparities across the PrEP care continuum, with PrEP non-adherence rates consistently observed among Black compared to White persons.^18^, ^19^, ^20^, ^21^, ^22^ In the United States, Black MSM had three times the odds of PrEP non-adherence compared to White MSM.^18^ This disparity is attributed in part to stigma, inadequate support systems, and financial barriers among Black MSM.^21^ A study in Chicago demonstrated significant attrition in PrEP retention among young Black individuals, highlighting modifiable barriers like clinic accessibility, insurance coverage, and intersectional stigma as contributors to these disparities.^22^

Worse PrEP outcomes among Black and *Pardo* participants are consistent with broader racialized social inequalities that continue to shape HIV prevention efforts in Brazil.^10^ Brazil is marked by profound socioeconomic disparities, in which race remains a central organizing axis of inequality.^23^ Systemic racism in Brazil has been linked to cumulative disadvantages across the life course, including poorer educational and employment opportunities, substandard housing, lower wages, inadequate healthcare, psychosocial stress, political marginalization, environmental injustices, and exposure to violence.^24^ These structural conditions may constrain engagement with biomedical prevention by producing competing priorities for food security, shelter, employment, and other health needs, which adversely affect PrEP use in Brazil.^25^ Black persons are disproportionally affected by these barriers.^26^ Socioeconomic disadvantages, compounded by systemic racism, may explain the higher rates of transactional sex among Black and *Pardo* compared to White participants, a behavior associated with PrEP non-adherence in our study. These dynamics likely reflect structural constraints rather than individual behavioral choices alone, and highlight how intersecting dimensions of inequality such as poverty, schooling, and structural discrimination may shape HIV prevention outcomes and PrEP use.

Racism also compromises access to health care and has been consistently associated with worse health outcomes for Black and *Pardo* compared to White Brazilians.^11^^,^^27^ Black and *Pardo* individuals are less likely to attend health services periodically^28^ and often suffer discrimination by healthcare professionals, which discourages them from seeking medical care.^29^^,^^30^ Geographic factors further compound these inequities, as many Black and *Pardo* Brazilians live in peripheral territories characterized by reduced availability of health services and limited institutional resources. In addition, stigma associated with HIV and racialized marginalization may deter individuals from seeking prevention and care. Fear of discrimination and mistrust in health institutions can lead to delayed testing or treatment, exacerbating health outcomes for those affected by HIV.^10^

Disparities in HIV incidence and PrEP non-adherence cannot be fully understood through individual behavior or logistical barriers alone. Black men have not merely been neglected from care; they have been historically positioned outside the boundaries of legitimate care.^31^ In this regard, Black masculinities in Brazil must be considered as a critical analytical lens. The historical and social construction of Black masculinity shaped by systemic dehumanization, criminalization, and institutional exclusion has contributed to the internalization of a symbolic condition of non-entitlement to rights.^32^, ^33^, ^34^ Rooted in colonial legacies that persist in contemporary modern health systems and sustained by racialized modernity, the current landscape situates Black men as structural “non-subjects”, a position where access to dignity, bodily autonomy, and full citizenship is persistently denied.^34^, ^35^, ^36^ The coloniality of power not only racialized Black bodies but also established sociopolitical dynamics that operate simultaneously at institutional and subjective levels, producing an ongoing, symbolic disconnection between Black male persons and the health care system.^37^, ^38^, ^39^

Despite being a cornerstone of biomedical HIV prevention, PrEP remains largely disconnected from Black and *Pardo* individuals residing in socioeconomically marginalized and racialized territories. While eligibility frameworks are nominally universal, structural racism and systemic inequities compromise their effective reach, producing patterns of exclusion and underutilization among Black and *Pardo* populations. A prior qualitative study from Brazil showed that Black MSM perceived PrEP as a distant or unrelatable intervention, often implicitly racialized as being for “other bodies”, such as White, gay, urban, affluent, and those well connected to social networks.^40^ To address these inequities, targeted interventions are urgently needed to mitigate the social determinants of health affecting PrEP adherence. The National Policy for the Integral Health of the Black Population, launched in Brazil in 2009,^41^ aims to reduce inequalities in the Brazilian Public Health System. However, only 1,781 (32%) of Brazilian municipalities had implemented these policies in their municipal health plans by 2021. These findings highlight persistent gaps between policy formulation and local-level execution, which may limit the capacity of health systems to equitably support PrEP use and adherence among Black and *Pardo* populations.

Regardless of race, participants of younger age and self-identifying as transgender women had increased odds of PrEP non-adherence. This is worrisome as these populations are disproportionately affected by HIV and may experience compounding barriers to PrEP adherence.^2^^,^^3^ For transgender women, intersectional stigma (transphobia alongside racism), concerns about gender-affirming care, and negative experiences in health services can erode trust and continuity in HIV prevention services, increasing PrEP adherence.^42^ Among younger MSM, greater life instability (housing, work, schooling), lower autonomy in navigating health systems, and fear of disclosing sexual orientation are potential barriers to maintain regular routines and follow-up visits required for PrEP.^43^^,^^44^ Taken together, these results underscore an urgent need for tailored, affirming, youth-friendly and trans-inclusive PrEP services. Effective strategies to support PrEP adherence, such as mobile text messages and peer support,^44^^,^^45^ should be implemented for these populations. Furthermore, strategies aimed at mitigating structural barriers, including differentiated service delivery models, stigma reduction within healthcare settings, and psychosocial support, should be considered.

The scarcity of evidence on PrEP adherence among Black individuals outside the United States underscores the significance of this study. With 3,928 participants from multiple clinics and regions form Brazil, the study provides a robust and diverse dataset, enhancing the reliability of its findings. Moreover, the results have clear policy implications by highlighting systemic inequities and emphasizing the need for targeted public health interventions to address these disparities effectively. However, this study has limitations. Participants were recruited in specialized HIV/STI clinics, which may limit generalizability to persons not engaged in healthcare. Sexual behavior and substance use were self-reported, which may be affected by recall bias and social desirability bias. The wide confidence intervals for HIV incidence rates reflect the limited number of events, resulting in some instability in the estimates. This includes strata with zero events, for which confidence intervals are inherently imprecise. Nevertheless, this pattern is expected in PrEP implementation cohorts, where overall HIV incidence is low due to effective prevention.^46^^,^^47^ PrEP adherence was assessed using MPR, which does not confirm actual pill intake and may overestimate adherence. However, previous analyses from the PrEP Brasil study indicate that MPR is a valid indirect adherence measure for distinguishing participants with protective tenofovir diphosphate concentrations from those without.^48^ Additionally, we found accuracy of MPR in discriminate protective tenofovir diphosphate concentrations among 4,257 Dry Blood Spot (DBS) samples of 2,096 participants (1692 MSM aged 18-years and 404 transgender women) of the ImPrEP study.^49^ The study questionnaire did not allow direct measurement of factors identified in the literature as important for PrEP non-adherence, such as PrEP awareness, stigma and inadequate social support systems. Association of behavior factors such as binge drinking and condomless anal sex with PrEP non-adherence may be affected by unmeasured cofounders, such as HIV risk perception.

In conclusion, our findings revealed significant racial and social disparities in PrEP non-adherence among MSM and transgender women in Brazil. These disparities contributed to higher HIV incidence among Black and *Pardo* Brazilians compared to White Brazilians. Biomedical technologies alone may not fix the gaps in HIV prevention, especially when they are not connected to anti-racist and community-led strategies. Achieving equity in HIV prevention requires more than just rhetoric. There is an urgent need for new strategies in healthcare systems that include anti-racist education, fair data practices, community-focused policies, and an approach that sees Black and *Pardo* not just as recipients of care but also as persons who are leaders and rights-holders.

## Ethics approval and consent to participate

The study is registered in the Brazilian Registry of Clinical Trials, U1111-1217-6021. The study was approved by the institutional review board of the Instituto Nacional de Infectologia Evandro Chagas, Fundação Oswaldo Cruz (INI-Fiocruz, Rio de Janeiro, Brazil; approval number CAAE 79259517.5.1001.5262) in accordance with the Resolution 466/12 of the Brazilian National Health Council. Ethics approvals were also obtained for WHO Research Ethics Review Committee and local institutional review boards at each Brazilian site. All participants provided written informed consent in Portuguese prior to any study procedure.

## Consent for publication

Not applicable.

## Availability of data and materials

A complete de-identified dataset sufficient to reproduce the study findings will be made available upon request to the corresponding author, following approval of a concept sheet summarizing the analyses to be done.

## ImPrEP study group

José Valdez Madruga, Alessandro Farias, Marcus Vinícius de Lacerda, Josué N Lima, Ronaldo Zonta, Lilian Lauria, J. David Urbaez-Brito, Polyana d’Albuquerque, Claudio Palombo, Paulo Ricardo de Alencastro, Raquel Keiko de Luca Ito, João L. de Benedetti, Fabio V. Maria, Paula M. Luz, Lucilene Freitas, Kim Geraldo, Monica Derrico, Sandro Nazer, Tania Kristic, Marcella Feitosa, Renato Girade (in memoriam), Renato Lima, Antônio R. de Carvalho, Carla Rocha, Pedro Leite, Marcio Lessa, Marilia Santini, Daniel R. B. Bezerra, Luana M. S. Marins, Cleo de Oliveira Souza, Jacinto Corrêa, Marcelo Alves, Carolina Souza, Camilla Portugal, Mônica dos Santos Valões, Gabriel Lima Mota, Joyce Alves Gomes, Cynthia Ferreira Lima Falcão, Fernanda Falcão Riberson, Luciano Melo, Talita Andrade Oliva, Agnaldo Moreira de Oliveira Júnior, Bruna Fonseca, Leonor Henriette de Lannoy, Ludymilla Anderson Santiago Carlos, João Paulo da Cunha, Sonia Maria de Alencastro Coracini, Thiago Oliveira Rodrigues, Emília Regina Scharf Mettrau, Kelly Vieira Meira; Heder Tavares, Ana Paula Nunes Viveiros Valeiras, Taiane Miyake Alves de Carvalho Rocha, Alex Amorim, Patrícia Sabadini, Luiz Gustavo Córdoba; Caio Gusmão, Erika Faustino, Julia Soares da Silva Hansen, Agatha Mirian Cunha, Neuza Uchiyama Nishimura, Jaime Eduardo Flygare Razo Prereira dos Santos, Aline Barnabé Cano, Willyam Magnum Telles Dias, Magô Tonhon, Tania Regina Rezende, Alex Gomes, Eloá dos Santos Rodrigues, Maria das Dores Aires Carneiro, Alexandre Castilho, Mariana Carvalho.

## ImPrEP study sites

Fundação de Medicina Tropical (Manaus, Amazonas), Hospital Universitário Oswaldo Cruz (Recife, Pernambuco), CEDAP – Centro Estadual Especializado em Diagnóstico, Assistência e Pesquisa (Salvador, Bahia), Hospital Dia Asa Sul (Brasília, Distrito Federal), Instituto Nacional de Infectologia Evandro Chagas, Fundação Oswaldo Cruz INI-Fiocruz (Rio de Janeiro), Hospital Municipal Rocha Maia (Rio de Janeiro), Hospital Municipal Carlos Tortelly (Niterói, Rio de Janeiro), Centro de Referência em DST/AIDS- AMDA (Campinas, São Paulo), Centro de Referência e Treinamento em DST/AIDS – CRT-SP (São Paulo), SAE DST/AIDS – CECI (São Paulo), SAE DST/AIDS – Fidélis Ribeiro (São Paulo), SAE Adulto (Santos, São Paulo), Poli Centro (Florianópolis, Santa Catarina), SAT – Sanatório Partenon (Porto Alegre, Rio Grande do Sul).

## Authors’ contributions

VGV, BG, and BH conceived and designed the ImPrEP study. LAF, CC, TST, BG and VGV conceived and supervised the current analysis and manuscript preparation. LAF, CC, DC and TST interpreted the findings and drafted the manuscript. RIM, ICL, and MC accessed and verified the data. RIM, PL, ICL, and MC did the statistical analyses. BH, MB, and CP helped with data acquisition and interpretation of the findings. JF, TA, LM and MSTS were involved in revising the manuscript for important intellectual content. All authors read and approved the final manuscript. TST and VGV had final responsibility for the decision to submit for publication.

## Funding

This project was made possible thanks to Unitaid’s funding and support. Unitaid accelerates access to innovative health products and lays the foundations for their scale-up by countries and partners. Unitaid is a hosted partnership of WHO. TST was financed in part by Conselho Nacional de Desenvolvimento Científico e Tecnológico (CNPq; #304417/2025-4 and #405558/2025-2) and Carlos Chagas Filho Foundation for Research Support in the State of Rio de Janeiro (FAPERJ; #E-26/201.270/2022). BG was financed in part by CNPq (#313265/2023-2) and FAPERJ (#E.26/200.946/2022).

## Data availability statement

The data that support the findings of this study are available from the corresponding author upon reasonable request.

## Conflicts of interest

The authors declare no conflicts of interest.
